# Feline myocardial transcriptome in health and in hypertrophic cardiomyopathy—A translational animal model for human disease

**DOI:** 10.1371/journal.pone.0283244

**Published:** 2023-03-16

**Authors:** Jessica Joshua, Jeff Caswell, M. Lynne O’Sullivan, Geoffrey Wood, Sonja Fonfara

**Affiliations:** 1 University of Guelph, Ontario Veterinary College, Department of Pathobiology, Guelph, Ontario, Canada; 2 University of Guelph, Ontario Veterinary College, Department of Clinical Studies, Guelph, Ontario, Canada; 3 University of Prince Edward Island, Department of Companion Animals, Charlottetown, Prince Edward Island, Canada; Mayo Clinic, UNITED STATES

## Abstract

Hypertrophic cardiomyopathy (HCM) is the most common heart disease in cats, characterized by primary left ventricular hypertrophy. Feline HCM closely resembles human HCM and is suggested as translational animal model for the human disease. A genetic cause is established in humans and suspected for cats, but little is known about the gene expression and pathways involved in the pathogenesis of HCM. To investigate the myocardial transcriptome changes in HCM, RNA sequencing was conducted on left ventricle (LV) and left atrium (LA) samples of healthy cats and cats with HCM (each n = 5; 20 samples). Ingenuity Pathway Analysis was used to determine functional pathways, regulators, and networks. Distinct gene expression profiles were identified in the LV and LA of the feline healthy and HCM myocardium. Analysis of differentially expressed mRNAs (>2 fold; FDR < 0.01) found chamber-specific (LV vs. LA) expression in both healthy and HCM groups, with higher transcriptional activity in the LA. Genes that contribute to the distinct structure and function of each chamber in health and HCM were identified in the regional comparison. The gene expression profiles of HCM compared to healthy hearts revealed disease related genes, including *THBS4* and *KLHL33* (LV), *FAM177B* and *THRSP* (LA), the latter 3 have not been reported for the myocardium so far, as the top differently expressed genes in the HCM heart. Differently expressed genes and functional pathways found in the HCM heart are associated with cardiac remodeling and fibrosis, inflammation, microvascular changes, calcium signaling and cardiac metabolism, with some regional differences. RhoGDI-RhoGTPase signaling, integrin and ILK signaling pathways, the LXR/RXR pathway in the LA, and the PPARα/RXRα, HIF1α and CXCR4 pathways in the LV might be of particular importance in the HCM disease process. This study identified region-specific myocardial gene transcription patterns as well as novel genes and pathways associated with HCM.

## Introduction

Hypertrophic cardiomyopathy (HCM) is the most prevalent cardiac disease in cats, affecting ~15% of the population [1, 2]. HCM is also common in humans affecting 1 in 500 people [3]. Spontaneous feline HCM closely resembles the human disease regarding phenotype, clinical presentation, and histological changes, and is suggested as translational model for human HCM [4–6]. The disease is characterized by primary left ventricular hypertrophy and diastolic dysfunction [4, 5] and is typically associated with variable clinical presentation and disease progression; many patients remain asymptomatic for years while others develop end-stage cardiomyopathy and severe outcomes such as congestive heart failure and sudden death [7–9]. Histopathological changes include cardiomyocyte degeneration, cardiomyocyte disarray, interstitial fibrosis, accumulation of inflammatory cells, and microvascular changes [5, 9–14]. This overall similarity of feline and human HCM suggests a comparable pathogenesis [4–6, 15]. A genetic cause of HCM is known for humans [16] and suspected for cats based on its prevalence in certain breeds [17–20] and the identification of mutations in sarcomeric genes in cats with HCM, similar to humans [17–19, 21–23]. Genetic variants have been detected in 2 sarcomeric genes using fragment analysis: p.A31P and p.R820W in myosin-binding protein C. These are associated with HCM in Maine Coon and Ragdoll cats, respectively [17, 18, 23]. A p.E1883K variant in the myosin heavy chain 7 gene has been found in a domestic shorthair cat with HCM [19]. These HCM mutations are suspected to affect the function of the sarcomere [24]. Additionally, a non-sarcomeric mutation in the Alstrom syndrome 1 (ALMS1) gene has been identified in Sphynx cats with HCM [20]. However, regulatory factors and signaling pathways involved in the development and progression of HCM are mainly unknown.

Transcriptome profiling is used to determine the abundance of each messenger RNA (mRNA) transcript within a cell or tissue. The mapping of mRNA transcripts provides insight into gene activation and inhibition patterns under physiological conditions and with disease. Recent ‘omics’ research in human cardiomyopathies and particularly in HCM identified between 154 and 4161 differentially expressed genes (DEG) in myocardial samples from humans with HCM, based on the selected database, analysis method and filter criteria [25–31]. These studies revealed disease-specific gene expression in human patients with HCM. To explore the biological functions and pathways of the DEG, Gene Ontology (GO) and Kyoto Encyclopedia of Genes and Genomes (KEGG) pathway enrichment analysis are commonly used. For human HCM these tools identified pathways involved in protein synthesis, myocyte survival, mitochondrial energetics, fatty acid metabolism, apoptosis, extracellular matrix, sarcomere organization, intracellular calcium handling, autophagy and angiogenesis [25–30]. These studies used either left ventricular samples only or provided no regional information (‘myocardial samples’, ‘myocardial/heart tissue), patient sex was not considered in the analysis [28–31]. While a few studies have investigated gene expression in cats with HCM using reverse transcription quantitative polymerase chain reaction (RT-qPCR) or microarrays [10, 11, 22, 32–35], high-throughput RNA sequencing (RNA-seq) has so far not been used, hence knowledge on all DEG and pathways involved in feline HCM is still lacking.

Regional differences in gene activation have been identified for the healthy human and feline heart, and for cats with HCM [33–38]. We therefore hypothesized that the myocardial transcriptome differs between cardiac regions (left ventricle; LV and left atrium; LA) in both healthy cats and cats with HCM. We also hypothesized that the HCM gene expression profile differs from that of healthy cats and would uncover genes and pathways activated or inhibited regionally and in HCM. The objective of the present study was to apply next-generation sequencing technology to characterize the healthy and HCM transcriptome profiles and identify regional and disease associated differences in myocardial gene expression.

## Material and methods

### Myocardial samples

Samples from cats with HCM were available from a previous study [33, 34]. These hearts were obtained from pet cats (between 3 and 15 years of age; Table 1) with an advanced stage of HCM that had been patients of a cardiology referral service. The cats were assessed and HCM was diagnosed by a specialist in veterinary cardiology using echocardiography. A diastolic LV wall thickness > 6 mm in the absence of other diseases that might result in LV wall thickening was considered diagnostic for HCM [4]. Indirect blood pressure measurement and complete blood count and routine biochemistry was carried out to exclude systemic hypertension and systemic diseases as potential cause for LV hypertrophy. Most cats had been long-term patients of the cardiology service and progression of their disease over time was observed. The cats were euthanized for medical reasons, and cat owners provided informed written consent that the hearts could be used for research purposes. The Animal Care Committee of the University scrutinised the consent form and the information given to the owner about the project to ensure that they were fully informed that research would be conducted on tissues obtained post mortem. Heart tissue specimen from male healthy cats (1.5 years of age; Table 1) were donated to the study by a commercial company that carries out animal safety studies for Food and Drug Administration and European Medicines Agency regulatory submissions in support of veterinary drug development and is certified by the Canadian Council on Animal Care (https://kingfisherint.com; https://ccac.ca/en/about-the-ccac/). The samples were collected from a healthy control group of their research cat population; these cats were considered healthy based on physical examination, complete blood count and routine biochemistry carried out by a veterinarian prior to euthanasia and post mortem examination.

**Table 1:** Cats included in the study and statistics summary of RNA sequencing data.

Sample	Group	Age (years)	Breed	Sex	Read pairs post trimming, n	Ribosomal RNA, %	Mitochondrial RNA, %	Uniquely mapped read pairs, %	Reads mapped to same strand, %	Reads mapped to opposite strand, %	Reads with not determined strand, %
**LV1**	Healthy LV	1.5	DSH	Male	47,478,136	0.03	19.55	76.09	91.55	2.30	6.15
**LV2**	Healthy LV	1.5	DSH	Male	43,888,937	0.03	21.93	71.16	92.42	2.48	5.10
**LV3**	Healthy LV	1.5	DSH	Male	44,123,313	0.03	18.25	74.39	91.12	2.50	6.39
**LV4**	Healthy LV	1.5	DSH	Male	42,265,613	0.03	18.10	74.32	91.80	2.40	5.80
**LV5**	Healthy LV	1.5	DSH	Male	49,505,256	0.03	18.30	73.29	90.90	2.49	6.61
**LV6**	HCM LV	9	DSH	Male	43,888,937	0.03	21.93	71.16	92.42	2.48	5.10
**LV7**	HCM LV	14	DSH	Male	44,077,351	0.03	17.35	71.55	90.74	2.59	6.67
**LV8**	HCM LV	15	DLH	Male	43,482,375	0.03	25.00	72.94	91.46	2.50	6.04
**LV9**	HCM LV	13	DSH	Male	48,884,797	0.03	13.67	81.88	89.75	2.56	7.69
**LV10**	HCM LV	3	DSH	Male	42,426,885	0.03	19.62	77.35	92.11	2.43	5.46
**LA1**	Healthy LA	1.5	DSH	Male	40,543,094	0.03	20.33	71.69	92.38	2.67	4.95
**LA2**	Healthy LA	1.5	DSH	Male	45,027,733	0.03	16.67	77.39	91.72	2.54	5.74
**LA3**	Healthy LA	1.5	DSH	Male	46,014,375	0.03	16.57	74.57	91.20	2.48	6.32
**LA4**	Healthy LA	1.5	DSH	Male	40,878,409	0.03	18.18	77.44	92.21	2.30	5.49
**LA5**	Healthy LA	1.5	DSH	Male	46,098,081	0.03	15.64	78.41	91.10	2.61	6.29
**LA6**	HCM LA	9	DSH	Male	51,695,982	0.03	15.37	78.91	88.56	2.36	9.09
**LA7**	HCM LA	14	DSH	Male	51,877,661	0.04	13.18	63.02	90.69	2.49	6.82
**LA8**	HCM LA	15	DLH	Male	46,463,111	0.04	16.17	76.02	91.75	2.37	5.89
**LA9**	HCM LA	12	DLH	Male	41,948,835	0.04	19.49	72.29	90.70	3.51	5.79
**LA10**	HCM LA	3	Ragdoll	Male	50,189,962	0.03	12.69	80.01	90.71	2.52	6.76

Left ventricle (LV) and left atrium (LA) samples from healthy cats and cats with hypertrophic cardiomyopathy (HCM). Domestic Shorthair (DSH), Domestic Longhair (DLH) and Ragdoll cats were included in the study

The inclusion criteria for the study were the presence of HCM in cats diagnosed with HCM and the absence of cardiac and systemic diseases in the control cats. To ensure that these inclusion criteria were met, gross and histopathology examinations were conducted and supervised by a board-certified specialist in veterinary pathology as the gold standard in all cats to confirm HCM in the cats clinically diagnosed with the disease, the absence of cardiac diseases in the healthy cats and the absence of systemic diseases in all cats. As previously published, HCM criteria comprised of increased left ventricular wall thickness, cardiomyocyte degeneration, cardiomyocyte disarray, interstitial fibrosis, accumulation of inflammatory cells, and microvascular changes [10–14]. Only hearts from male cats were included to limit the potential influence of sex on the results, since sex differences in myocardial gene expression have been reported for non-failing human hearts, healthy cats, and cats with HCM [34, 36, 38]. Furthermore, to ensure appropriate RNA quality, only samples collected within 30 minutes after death were included into this prospective observational post mortem study. Feline heart specimens were examined utilizing RNA sequencing (RNA-seq) to detect and quantify all the expressed mRNA transcripts. In total, 20 samples were included: 5 LV and 5 LA samples of male cats with HCM and 5 LV and 5 LA of male healthy cats (Table 1). LV free wall and LA samples were collected immediately after euthanasia and were stored in RNAlater at room temperature for 24 hours, followed by storage at -80 °C until further use.

### RNA isolation and quality control

For extraction of total RNA, 700 μL of QIAzol Lysis Reagent (Qiagen, Toronto, ON, Canada) and a 2 mm stainless steel bead were added to myocardial tissue and lysed with using Tissue Lyser II (Qiagen, Toronto, ON, Canada) for 8 minutes at 30/s frequency for high throughput sample disruption and homogenization. The miRNeasy Mini Kit (Qiagen, Toronto, ON, Canada) was subsequently used to isolate total RNA, according to manufacturer’s protocol from 20 samples: 5 LV and 5 LA samples of healthy cats and 5 LV and 5 LA of cats with HCM. In general, RNA was purified from tissue using spin columns and a series of wash buffers to bind RNA to the silica membrane, which was then eluted in RNase free water. RNA purity was assessed using the Nanodrop 2000 spectrophotometer (Thermo Scientific, DE, USA). RNA integrity was measured using the Agilent Bioanalyzer 2100 system (Agilent Technologies, CA, USA). Samples with a 260/280 ratio of >1.9 and RNA integrity number of >5.7 were used for sequencing. Extracted RNA samples were stored at -80 °C until used for sequencing.

### Library preparation and sequencing

A stranded, paired-end poly-A mRNA library was prepared for each sample. Sequencing libraries were generated using NEBNext Ultra II Directional RNA Library Prep Kit for Illumina (New England Biolabs, USA) as per manufacturer’s instructions. Briefly, mRNA was isolated, fragmented and primed from total RNA. Then, the first strand and second strand cDNA synthesis were performed. After, the cDNA fragments were ligated to adapters and underwent PCR amplification according to Illumina instructions. Library quality was assessed using the Bioanalyzer 2100 system (Agilent Technologies). The libraries were sequenced on the NovaSeq 6000 system, SP flowcell 2 x 150 bp (Illumina). The *Felis_Catus_9*.*0*.*96* (Ensembl gene models) with annotations was used as the reference genome. RNA isolation and library preparation for all samples were performed in the same batch.

### Read pre-processing, read alignment and transcript assembly

Expression levels were represented by the number of reads of each transcript. After sequencing, the quality of the data was assessed using FastQC v.0.11.5 (http://www.bioinformatics.babraham.ac.uk/projects/fastqc). The adaptors were trimmed using Trim Galore v.0.4.4. Trimming was stopped if quality of base was greater than 25. The first 6 nucleotides were clipped from the 5’ ends and any read that was shorter than 40 nucleotides was discarded, with only pairs of reads retained. After trimming, the quality of reads was re-assessed with FastQC. Ribosomal RNA and mitochondrial RNA were screened using FastQ-Screen. RSeQC v.3.6.7 was used to assess read distribution, positional read duplication (read_distibution.py) and strandedness of the alignments (infer_experiment.py). 80% of the reads mapped to the exonic sequences. Raw trimmed reads were aligned to reference genome using the STAR aligner, followed by HTSeq-count v.0.6.1p2 to obtain uniquely mapped gene counts.

### Data normalization

Raw gene counts were normalized using DESeq v.1.18.0 (R package, Vienna, Austria). The amount of total RNA varied between samples and those with more RNA tended to have higher read counts for genes. For differential gene expression analysis, data were normalized using the trimmed mean of M-values method to avoid obtaining results caused by differences in total RNA between samples rather than the true variance in gene transcription.

### Principal component analysis

Principal Component Analysis (PCA) and hierarchical clustering were used to visualize genetic relationships by grouping samples together based on their similarities. PCA is an algorithm that determines the important variables, in the form of principal components (PC1, PC2) from a large set of variables in the data. All filtered transcripts were used to generate the PCA plot.

### Differential gene expression analysis

Following quantification, the identification of DEG between HCM and samples from healthy cats as well as between the LV and LA was performed using edgeR R package v.3.22.3 (http://www.bioconductor.org/packages/release/bioc/html/edgeR.html). Statistical significance was determined using a moderated t-test with the P-value corrected using the Benjamini-Hochberg algorithm. Genes were considered differentially expressed if they had log_2_ fold change ± 1 and a false discovery rate (FDR) < 0.01. Moreover, genes that showed less than 3 fragments per kilobase of transcript per million mapped reads in both sample groups were excluded, because the error of quantification is high at very low expression levels.

### Pathway and network analysis

To determine DEG and discover main functions and pathways, DESeq-identified transcripts were filtered based on statistical significance (log_2_ fold change ± 1, FDR < 0.01). Ingenuity Pathway Analysis (IPA, Qiagen Redwood City, CA [39]) was applied for downstream analysis. Expression core analysis was run to obtain gene orthologues as found in human, mouse and rat databases and to determine orthologous canonical pathways and networks from the data, considering both direct and indirect relationships using the IPA Knowledge Base. Also, sequencing data were mapped as graphical summaries to outline the most significant interactions among pathways, upstream regulators and biological processes as found by the IPA core analysis. As a predicted function, the Z-score (the number of standard deviations the data above or below the mean) indicated the activation (z > 0) or inhibition (z < 0) of genes or pathways. Cut-offs were P-value < 0.01 and log_2_ fold change ± 1.

### Validation by reverse transcription quantitative polymerase chain reaction (RT-qPCR)

To validate the sequencing data, several genes were selected to be verified by RT-qPCR, based on being among the most highly expressed in the HCM heart. 5 LV and 5 LA samples were collected from healthy cats and cats with HCM (n = 20 total). Total RNA was extracted (same protocol as RNA isolation above) and cDNA was synthesized by reverse transcription using Invitrogen Superscript III Reverse Transcriptase (ThermoFisher), according to manufacturer’s instructions. RT-qPCR was performed using the LightCycler 480 (Roche Diagnostics). PCR primer sequences (S1 Table) were selected using Primer Express v.3.0.1 for *CXCL14*, *CXCL6*, *IL18*, *THBS4*, *ANKRD2*, and *ID3*. Conditions for RT were as follows: 65°C for 5 mins, 50°C for 60 mins, 70°C for 15 mins and 37°C for 20 mins. The cycling conditions for qPCR were: 95 °C for 7 minutes, 45 cycles of 95 °C for 20 seconds, 60 °C for 20 seconds, 72 °C for 20 seconds, followed by dissociation curve analysis. *GAPDH* and *RSP7* (S1 Table) were used as housekeeping genes [40]. Relative quantification was calculated by the 2^-ΔΔCt^ method [41].

### Statistical analysis

The statistical significance of pathway enrichment was analyzed by using the hypergeometric distribution. False discovery rate was used as an adjusted P-value using the Bonferroni correction. All RT-qPCR statistical analyses were performed using SPSS (SPSS, Inc, IBM company, New York). RT-qPCR results were not normally distributed, and Kruskal-Wallis test was applied for comparison of groups: healthy LV vs. HCM LV, and healthy LA vs. HCM LA. P-values < 0.05 were considered as statistically significant.

## Results

### Characterization of the feline LV and LA transcriptome

To determine the feline myocardial transcriptome profiles of the healthy feline LV and LA and to identify changes associated with HCM as the most prevalent cardiac disease in cats, RNA-seq libraries were constructed from each 5 healthy (5 LV, 5 LA) and HCM (5 LV, 5 LA) myocardial samples. These included paired LV and LA samples from all healthy and 3 HCM cat hearts, and 2 LV and 2 LA from 4 different HCM hearts. The samples were selected based on quality control results (Nanodrop and Bioanalyzer). Demographic information of the cats is provided in Table 1. On average, 46.5 million read pairs were generated from each sample, and 34.6 million reads per sample aligned to the cat (*Felis catus*) reference genome (Table 1). In total, 82% of total mapped reads were uniquely mapped, of which 91% of reads were mapped to the same (sense) strand of the reference genome. The remaining reads (18%) were from other non-coding RNA, particularly mitochondrial RNAs. Only 11.9% of reads were unaligned to the annotated genome and 6.4% of sequences were reads without a determined strand, indicating potential novel mRNAs (Table 1).

**Fig 1:**
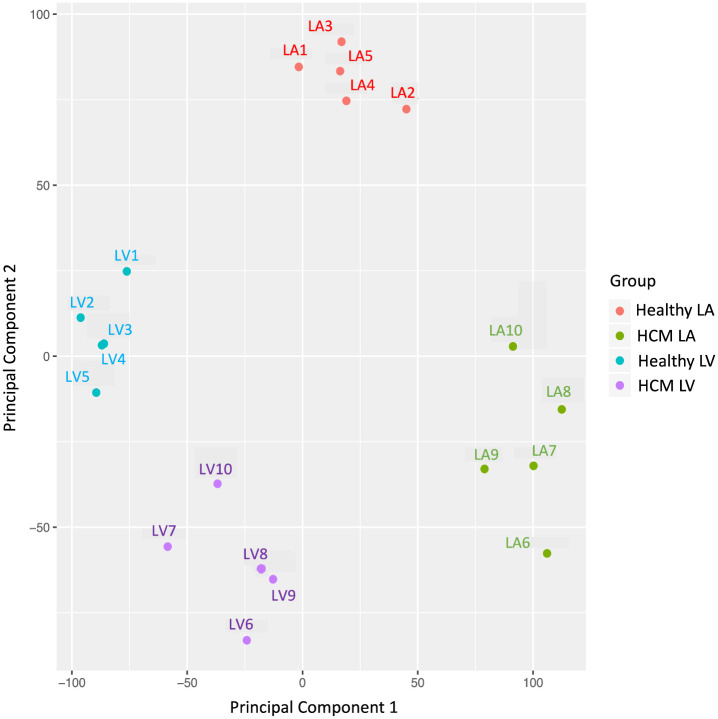
Left ventricle (LV) and left atrial (LA) samples have distinct mRNA profiles in healthy and HCM cats. Principal component analysis of all reads shows clustering of samples based on region (LV vs. LA) and disease status (healthy vs. HCM). The clustering of expression profiles within the feline heart distinguishes LV from LA samples along principal component 1 (PC1), and HCM from healthy myocardial samples along principal component 2 (PC2).

PCA was employed to cluster samples based on similarity of gene expression levels. This revealed low intragroup variability and high intergroup variability indicating that the transcriptomes were similar within each group and distinct between groups (Fig 1). Samples clustered along the first component (PC1) based on heart chamber (LV vs. LA) and along the second component (PC2) based on disease status (healthy vs. HCM). When comparing LV and LA samples of healthy and HCM hearts, the primary sources of variance in the RNA-seq data, as signified by the principal components, were strong regional and disease-associated mRNA expression differences (Fig 1).

### Region specific mRNA expression profiles, functional pathways and networks

#### mRNA expression profiles distinguish the healthy LV and LA, and the HCM LV and LA

**Fig 2:**
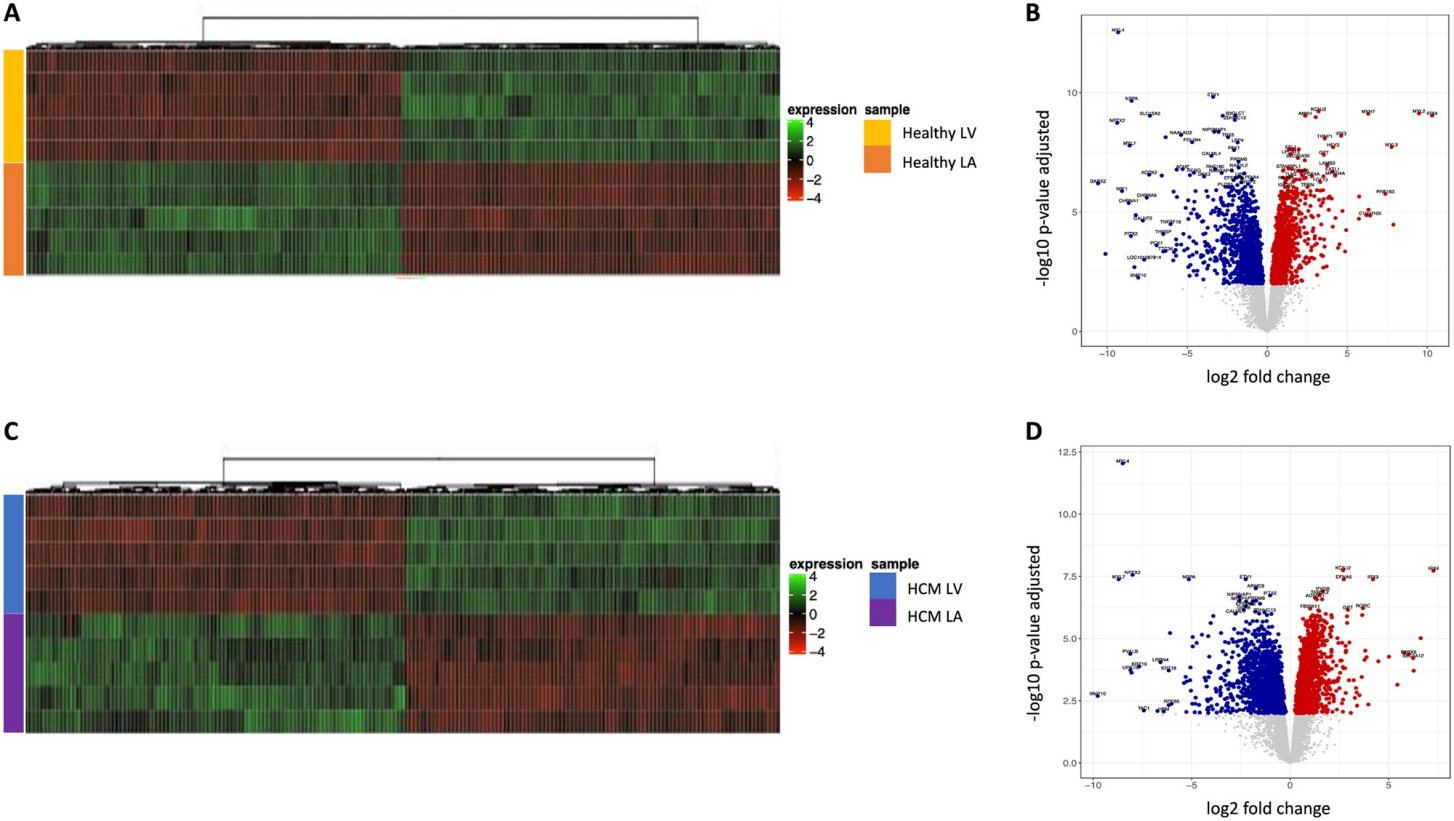
Differentially expressed mRNA transcripts distinguish left ventricle (LV) and left atrium (LA) in hearts from healthy cats and those with HCM. Heatmap of the 500 most differentially expressed mRNAs and volcano plots distinguish the LV from the LA samples in the healthy heart (A and B respectively) and the HCM heart (C and D respectively). Normalized read counts were filtered with the parameters Log2 Fold Change (Log_2_ FC) +/-1 and False Discovery Rate (FDR) < 0.01. Sample groups: healthy left ventricle (Healthy LV; yellow), healthy left atrium (Healthy LA; orange), HCM left ventricle (HCM LV; blue) and HCM left atrium (HCM LA; purple).

To identify regional differences in the transcriptome of the healthy heart, the mRNA profiles of the LV and LA from healthy cats were compared (Fig 2A and 2B). This was followed by a regional comparison, i.e. LV and LA of the feline HCM heart (Fig 2C and 2D). Heat maps were used to illustrate unsupervised hierarchical clustering of the normalized expression of the top 500 DEG across samples (Fig 2A and 2C). Volcano plots served to graphically display the most relevant genes by comparing statistical significance or the P-value against fold change (Fig 2B and 2C).

Healthy LV vs. healthy LA: A heat map of the healthy feline heart showed full separation of gene expression between the LV and the LA among the DEG (Fig 2A). The volcano plot revealed 1423 DEG in the healthy heart, 507 genes were expressed more abundantly and 916 genes less abundantly in the LV compared to the LA (Fig 2B). The top 25 genes with higher expression in the LV vs. LA included those known to be involved in sarcomere function (*MYL2*, *MYL3*, *MYH7*) and transcription factors (*Iroquois homeobox [IRX]4*, *IRX3*, *IRX2*) (S2A Table). The genes with lower expression in LV vs. LA included transcription factors (*BARX2*, *PITX2*), sarcomere genes (*MYL4*, *MYL7*), cholinergic receptors (*CHRNA1*, *CHRNA6*), and natriuretic peptide and its receptor (*NPPA*, *NPR3*) (S2B Table).

HCM LV vs. HCM LA: Regional differences were also observed in the myocardium of cats with HCM. A heat map of the HCM heart displayed a transcriptional profile of the LV that is distinct from the LA (Fig 2C). The volcano plot identified 1513 DEG in the HCM heart, 545 genes were expressed more abundantly, and 968 genes were less abundantly in the LV compared to the LA (Fig 2D). Among these were several genes that had also been found to be differently expressed in the healthy heart compartments. The top 25 genes with increased transcription in the LV vs. LA of cats with HCM included genes involved in nicotinamide adenine nucleotide metabolism (*ART5*, *ART1*) and transcription factors (*IRX4*, *IRX5*, *IRX3*) (S3A Table). The top 25 genes with reduced transcription in the LV vs. LA included sarcomere genes (*MYL7*, *MYL4*), epithelial cell and extracellular matrix genes (*COMP*, *WIF1*, *KRT7*, *KRT8*, *KRT18*) and natriuretic peptide (*NPPA*) (S3B Table).

#### Pathway signaling and regulators are activated in the LA compared to the LV in both healthy and HCM hearts

**Fig 3:**
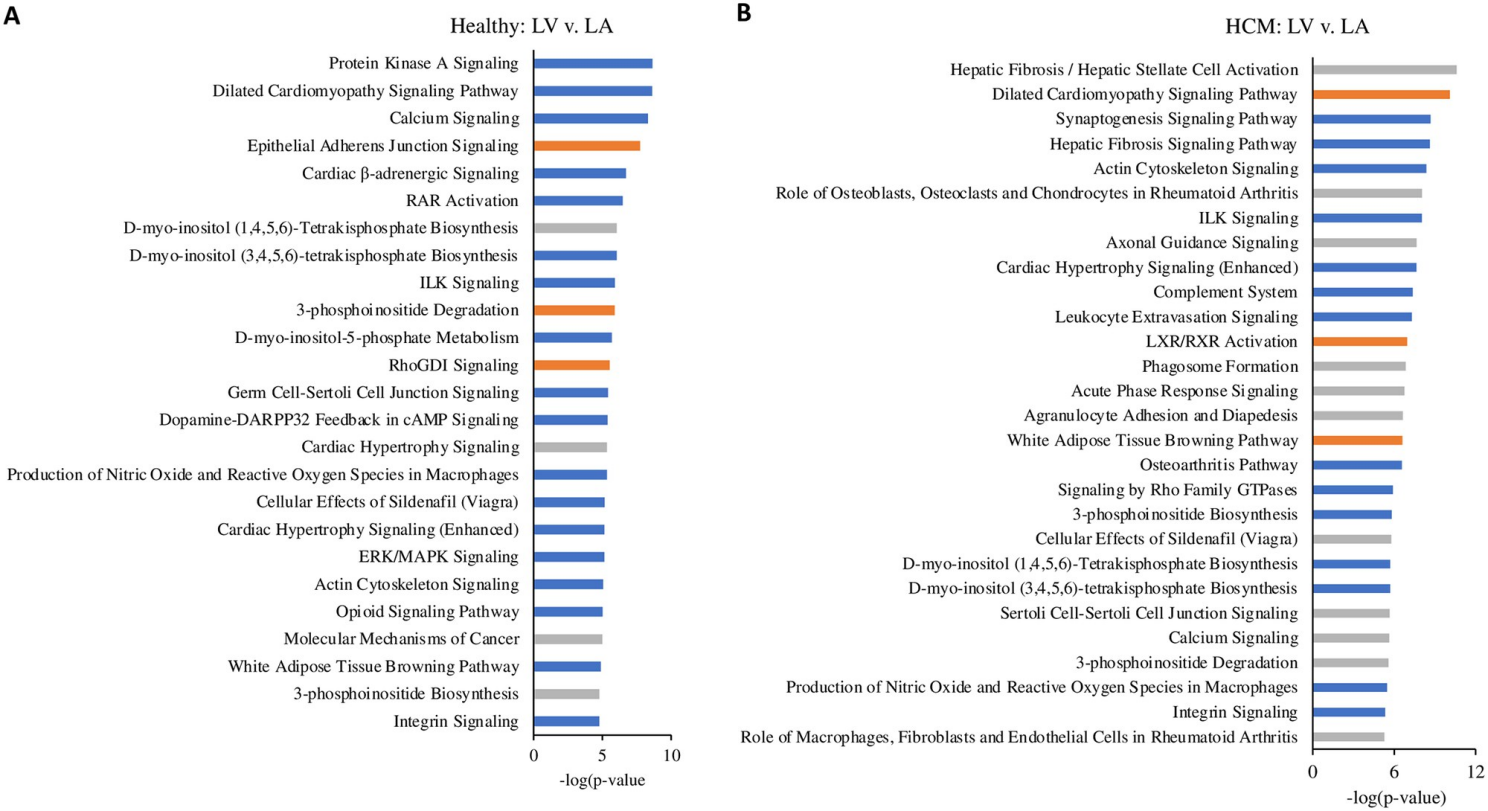
Top canonical pathways in the left ventricle (LV) compared to the left atrium (LA). The biological and disease pathways most affected by the altered gene expression levels in the LV compared to the LA, based on analysis of healthy hearts (A) and hearts from cats with HCM (B). All genes in the analysis were filtered P-value <0.01 and their positive or negative expression was identified based on their Z-score. Line colors indicate significantly increased expression (orange), significantly decreased expression (blue), and no direction (gray).

**Fig 4:**
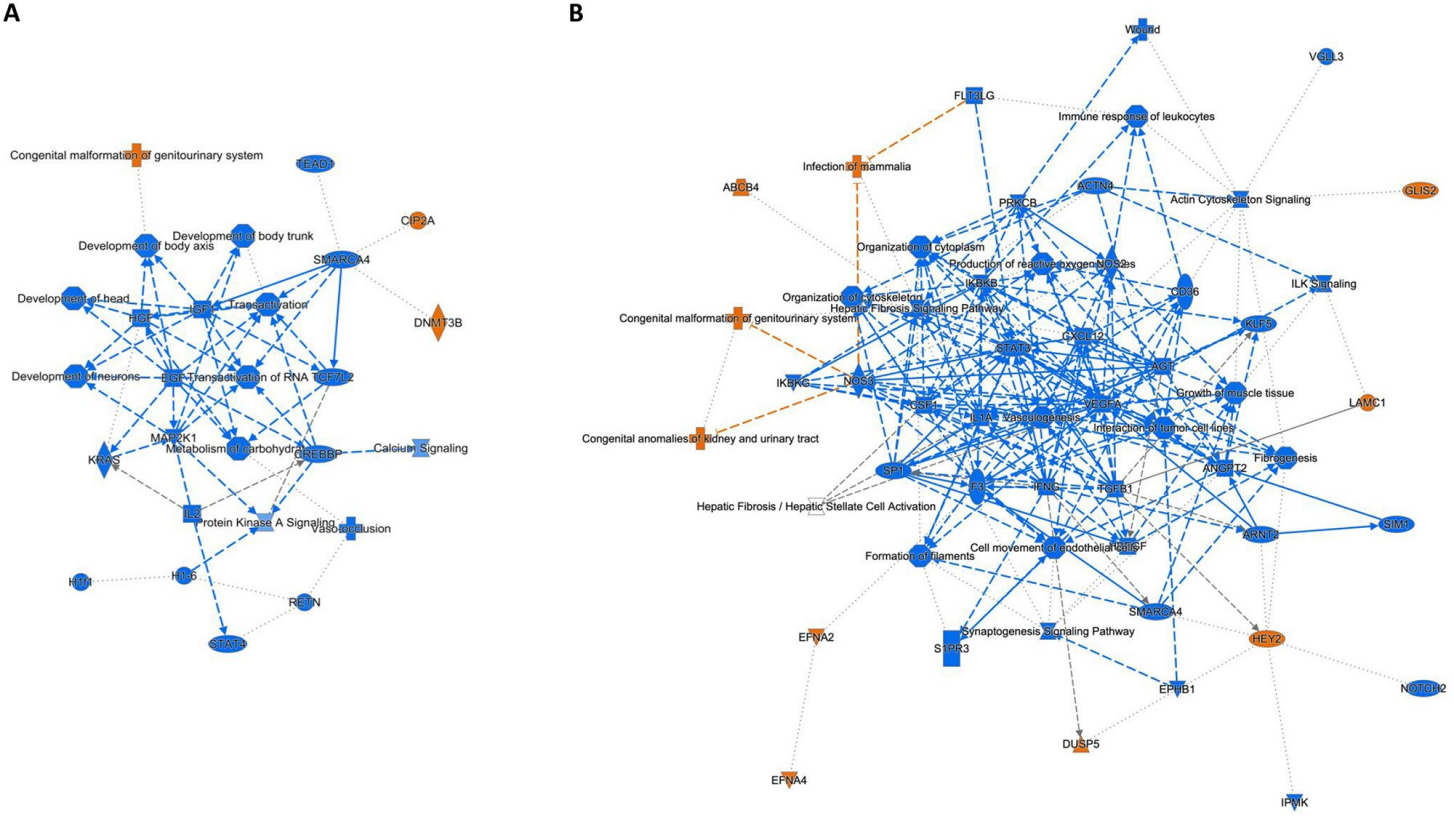
Network analysis in the left ventricle (LV) compared to left atrium (LA). Graphical summary of the IPA core analysis shows major pathways, regulators and processes within the LV compared to the LA based on analysis of hearts from healthy cats (A) and those with HCM (B). Network analysis shows interactions between differentially expressed genes within each group. Node and line colors indicate significantly increased expression (orange), significantly decreased expression (blue), and no direction (gray). For each function, z-scores were used to predict activation or inhibition. Lines and arrows between nodes represent direct interactions (solid) and indirect interactions (dashed) between molecules. Node shapes symbolize the functional class of genes: enzymes (diamond), phosphatases (triangle), complexes or groups (circle and hourglass), transcriptional regulators or modulators (oval) and cytokines (rectangle).

Next, we explored the biological functions of the DEG detected in the regional comparison of the healthy and HCM hearts. IPA was used to identify activated or inhibited major pathways, regulators and their interactions (Figs 3 and 4). For both the healthy and HCM hearts, IPA revealed that most pathways were activated in the LA compared to the respective LV (Figs 3A, 3B, 4A and 4B). For both HCM and healthy hearts, activated pathways in the LA included D-myo-inositol-phosphate biosynthesis, cardiac hypertrophy, actin cytoskeleton, ILK and integrin signaling as well as production of nitric oxide (NO) and reactive oxygen species (ROS) in macrophages (Fig 3A and 3B). The comparison of LV and LA in healthy and HCM cats found altered regional expression for genes of the dilated cardiomyopathy signaling pathway and the white adipose tissue browning pathway activation. These pathways were activated in the HCM LV and inhibited in the healthy LV when compared to the corresponding LA (Fig 3A and 3B). Top upstream regulators were TGFβ1 and beta-estradiol for both the healthy heart and HCM heart, and TNFα for the HCM heart. The IPA network analysis identified primarily developmental and metabolic pathways in the regional comparison for the healthy heart; fibrosis, structural, inflammatory and angiogenesis pathways were found in the regional comparison for the HCM heart (Fig 4A and 4B).

### HCM specific mRNA profiles, functional pathways and networks

#### mRNA expression profiles distinguish HCM and healthy hearts

**Fig 5:**
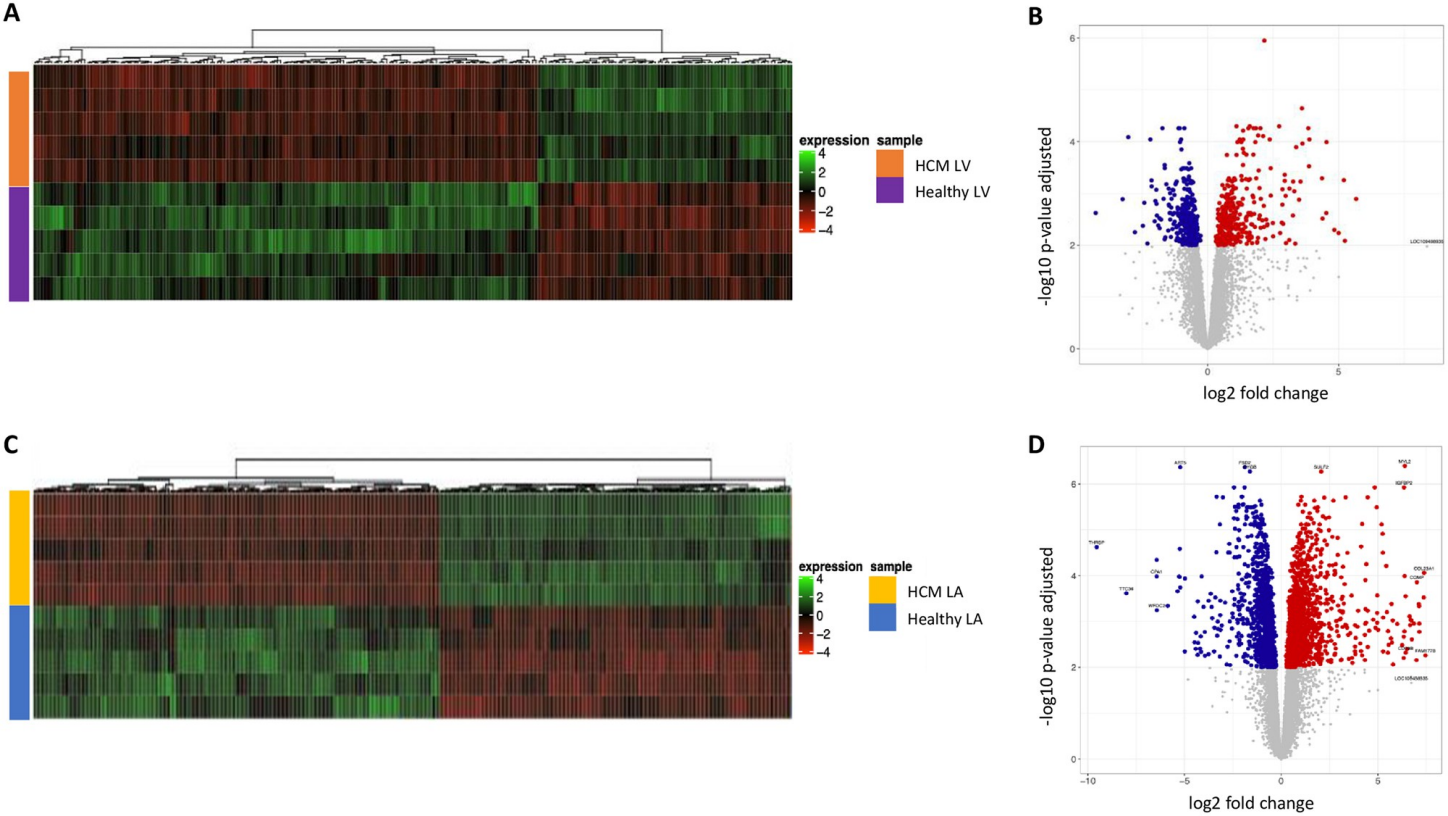
Differentially expressed mRNAs in the left ventricle (LV) and left atrium (LA) distinguish between the healthy and the HCM myocardium. Heatmaps of top 500 most differentially expressed mRNAs and volcano plots distinguish the healthy and HCM myocardial samples based on analysis of the LV (A and B respectively) and the LA (C and D respectively). Normalized read counts were filtered with the parameters Log_2_ FC +/-1 and FDR < 0.01. Sample groups: HCM left ventricle (HCM LV; blue), healthy left ventricle (Healthy LV; yellow), HCM left atrium (HCM LA; purple) and healthy left atrium (Healthy LA; orange).

To identify alterations in myocardial gene transcription in association with HCM, the LV and LA mRNA profiles of HCM cats were compared with those of their healthy counterparts. Heat maps and the volcano plots were used to visualize the differences (Fig 5A–5D).

HCM LV vs. healthy LV: The DEG showed a transcriptome profile that distinguishes the HCM LV from the healthy LV (Fig 5A and 5B). A total of 412 DEG were identified in the LV of HCM cats compared to the LV of healthy controls, 290 genes were upregulated and 122 downregulated in HCM (Fig 5B). The top 25 upregulated genes in the HCM LV included *THBS4* as the top upregulated gene, sarcomere genes (*MYL1*, *MYBPC2*) and genes involved in cell-to-cell interactions (*IGSF1*, *JCHAIN*), mechano-sensing (*SYNPR*, *ANKRD2*), remodeling and fibrosis (*GPNMB*, *LTBP2*, *UCHL1*, *FMOD*, *IGFBP2*, *OSTN*, *TNC*), and inflammation (*CXCL14*, *CXCL6*, *CCL19*, *IL18*) (S4A Table). The top 25 downregulated genes in the HCM LV included *KLHL33* (top downregulated gene), transcription factors (*ATF3*, *MYB*), *WNT5A* and *ART5* (S4B Table).

HCM LA vs. healthy LA: For the HCM LA, 1207 DEG were identified; of these 724 were upregulated and 483 downregulated in the HCM LA compared to the healthy LA (Fig 5C and 5D). Among the top 25 upregulated genes in the HCM LA, *FAM177B* had the highest enrichment. Sarcomere genes (*MYL2*, *MYL3*), extracellular matrix genes (*COL23A1*, *COMP*, *THBS4*), myocyte differentiation (*IGFBP2*), inflammation (*HP*, *CXCL6*, *CCL19*) and B- (*CD79A*) and T-cell surface glycoprotein (*CD3D*, *CD3G* and *CD3E*) genes were also higher in the HCM LA than healthy LA (S5A Table). Genes involved with fatty acid synthesis (*THRSP* [top downregulated gene], *DGAT2*, *FASN*, *ADIPOQ*), cardiac metabolism (*PFKFB1*, *ATF3*, *TRARG1*) and cell death activators (*CIDEA*, *CIDEC*) were among the top downregulated genes (S5B Table).

#### Pathway signaling and regulators are activated in the HCM heart

**Fig 6:**
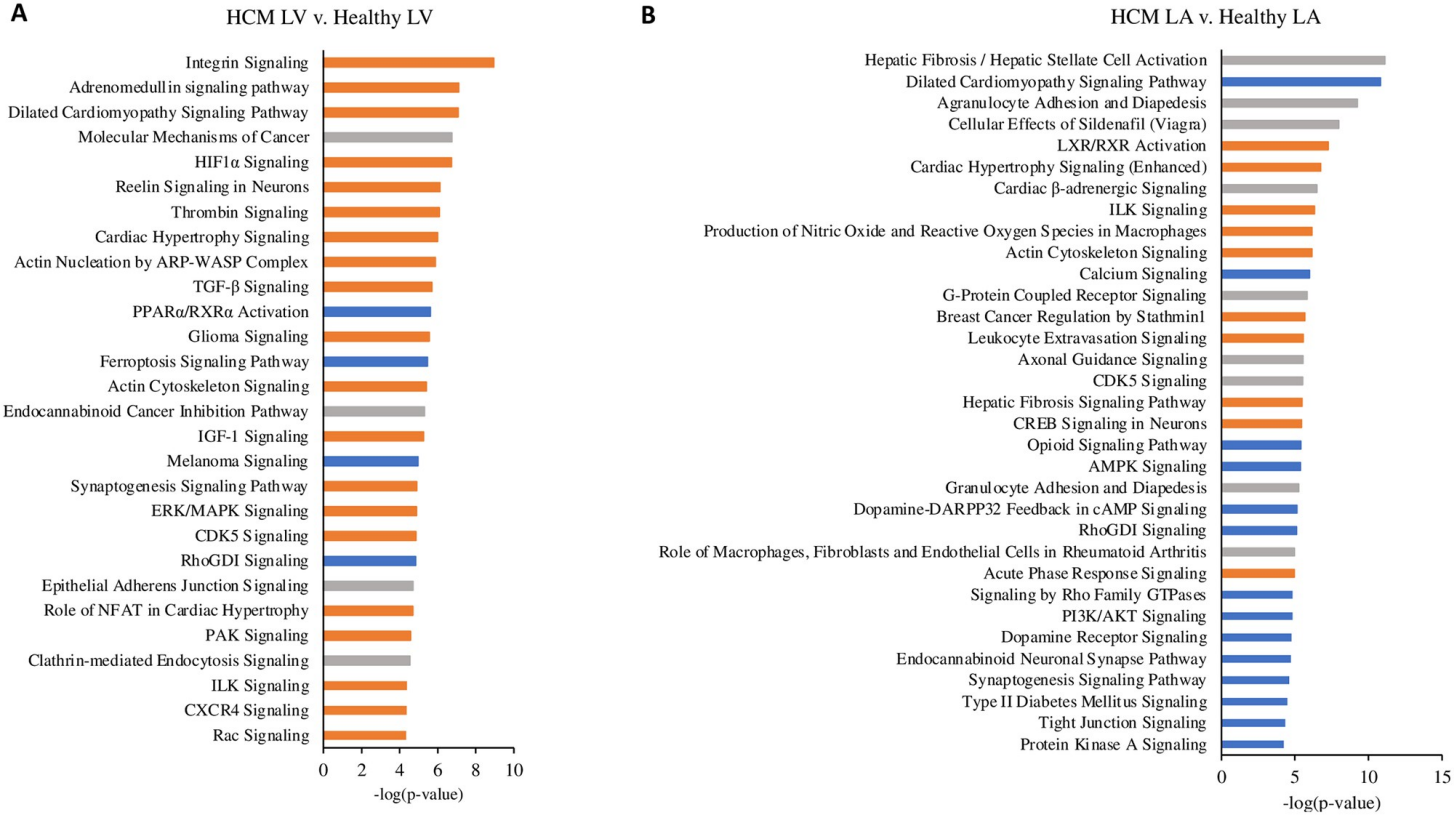
Top canonical pathways in the HCM heart compared to the healthy heart. The biological and disease pathways most affected by the altered gene expression levels in the left ventricle (LV: A) and left atrium (LA: B) of HCM cats relative to the LV and LA of healthy cats. All genes in the analysis were filtered P-value <0.01 and their positive or negative expression was identified based on their Z-score.

**Fig 7:**
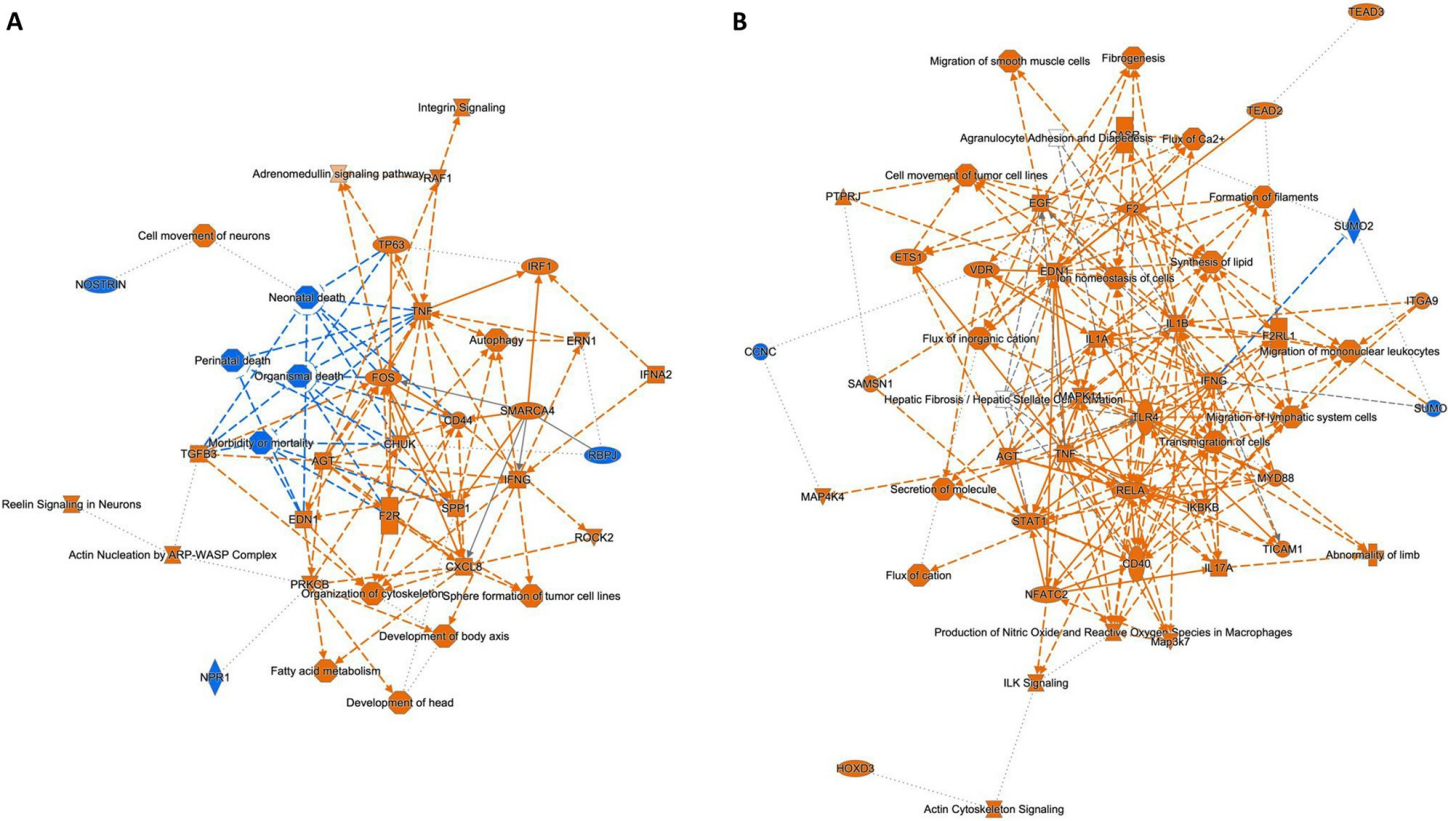
Network analysis in the HCM heart compared to the healthy heart. Graphical summary of the IPA core analysis shows major pathways, regulators and processes within the HCM left ventricle (A) and the HCM left atrium (B) that differs from their counterparts in the healthy heart. Network analysis shows interactions between differentially expressed genes within each group. Node and line colors indicate significantly increased expression (orange), significantly decreased expression (blue), and no direction (gray). For each function, z-scores were used to predict activation or inhibition. Lines and arrows between nodes represent direct interactions (solid) and indirect interactions (dashed) between molecules. Node shapes symbolize the functional class of genes: enzymes (diamond), phosphatases (triangle), complexes or groups (circle and hourglass), transcriptional regulators or modulators (oval) and cytokines (rectangle).

To explore the biological functions of the DEG observed in the HCM LV and LA, IPA and network analysis were applied and positively or negatively affected pathways and regulators were identified, along with their interactions (Figs 6A, 6B, 7A and 7B). The most activate canonical pathway in the HCM LV was integrin signaling (Fig 6A). Furthermore, signaling pathways associated with adrenomedullin, dilated cardiomyopathy, cardiac hypertrophy, actin nucleation and cytoskeleton, cardiac remodeling and inflammation, and ERK/MAPK were among the top activated pathways (Figs 6A and 7A). PPARα/RXRα activation, RhoGDI signaling, and death pathways were negatively affected in the HCM LV (Figs 6A and 7A). The top upstream regulator was TGFβ1.

Compared to the LV, fewer pathways were enriched in the HCM LA (Fig 6B). These included pathways related to LXR/RXR activation, cardiac hypertrophy signaling, ILK signaling, production of NO and ROS in macrophages, actin cytoskeleton signaling, fibrosis and cell migration pathways, and acute phase response signaling (Figs 6B and 7B). Dilated cardiomyopathy signaling, calcium signaling pathways, RhoGDI, Rho GTPase, PI3K/Akt and protein kinase A (PKA) signaling were negatively affected in the HCM LA (Fig 6B). The top upstream regulator was TNFα. Several pathways that have so far not been reported for the heart included: synaptogenesis (upregulated in the HCM LV and downregulated in the HCM LA), glioma and reelin (upregulated in the HCM LV) and stathmin 1 (upregulated in the HCM LA), as well as endocannabinoid neuronal synapse and opioid signaling (both downregulated in the HCM LA) (Fig 6A and 6B).

### RT-qPCR validation of RNA-seq results

**Fig 8:**
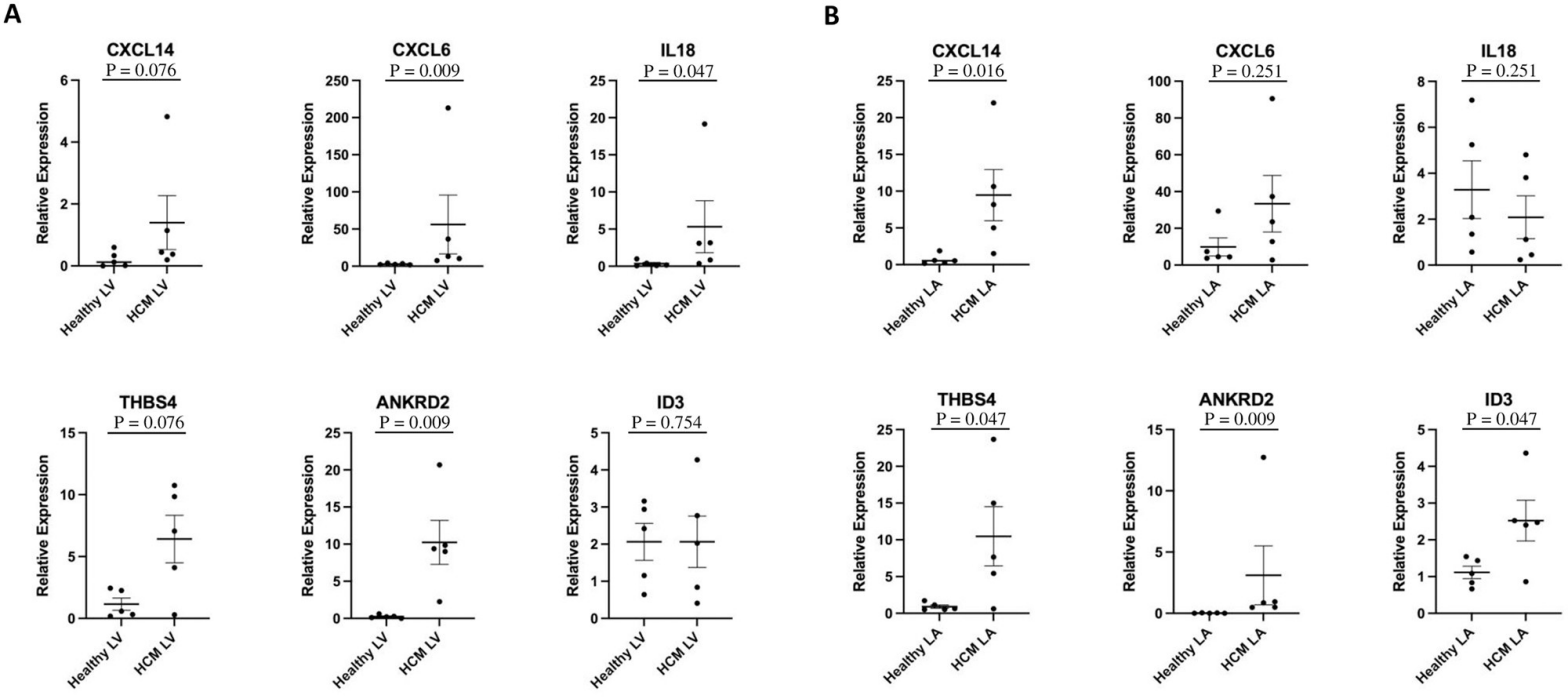
Validation of gene expression by qRT-PCR. HCM-associated differences in expression profiles in the left ventricle (LV: A) and left atrium (LA: B). Comparisons of mRNA levels determined by qRT-PCR show that *CXCL14*, *CXCL6*, *IL18*, *THBS4* and *ANKRD2* are expressed at higher levels in HCM LV compared to the healthy LV (A), and *CXCL14*, *THBS4*, *ANKRD2*, and *ID3* are expressed at higher levels in HCM LA compared to the healthy LA (B). Mean ± SEM (n = 5) values are indicated and the normalized expression values against reference genes *RSP7* and *GAPDH* are plotted.

To validate and confirm the sequencing results, RT-qPCRs were established for enriched genes of the HCM LV and LA: *CXLC14* (logFC HCM LV: 3.87, HCM LA: 2.17), *CXCL6* (logFC HCM LV: 3.49, HCM LA: 5.24), *THBS4* (log FC HCM LV: 5.67, HCM LA: 5.7), *ANKRD2* (logFC HCM LV: 3.34, HCM LA: 6.38), *IL18* (logFC HCM LV: 3.38), and *ID3* (logFC HCM LA: 1.04). Consistent with the sequencing data, *CXCL14*, *THBS4* and *ANKRD2* showed increased expression in the HCM LV and LA, *CXCL6* and *IL18* were higher in the HCM LV, and *ID3* in the HCM LA when compared to their healthy counterpart (Fig 8A and 8B).

## Discussion

The present study represents the first transcriptome analysis of the feline heart, identifying not only the constitutive transcriptome profile of the healthy LV and LA, but also its alterations in association with advanced HCM. RNA sequencing identified global changes in cardiac gene expression that distinguished the LV and the LA, as well as the presence of HCM in the feline heart. The biological function and pathways associated with the DEG revealed a unique gene expression pattern in the LV and LA that contribute to the distinct structure and function of each chamber in health and HCM. Regional differences have also been reported for the healthy human heart [36]. A total of 1423 and 1513 DEG were observed for the healthy feline heart and the HCM heart, respectively, comparing the LV and LA for each condition. Associated with HCM were 412 DEG for the LV and 1207 for the LA. The number of LV DEG is similar to that reported for human HCM based on RNA-seq [28, 31]. Several of the top regulated DEG, such as *THRBS4*, the top enriched HCM LV gene, sarcomere genes (*MYH*, *MYL)*, mechano-sensing (*ANKRD)*, fibrosis genes (*FMOD*, *COL*, *LTBP2*, *TNC*, *TGFβ)*, inflammation (*CXCL*, *CCL*, *IL*, *TNFα*), natriuretic peptides *(NPPA*, *NPR3)*, transcription factors (*IRX*, *STAT3)*, and predicted pathways and networks, including myocyte survival, fatty acid oxidation, cytoskeletal, fibrosis, mitochondrial function, calcium signaling, angiogenesis, in the HCM heart, have also been reported for human HCM hearts [27–31]. Interestingly, the top DEG *KLHL33* (LV), *FAM177B* and *THRSP* (LA) have not been reported in the heart so far and their role in HCM requires further investigation.

### Region-specific myocardial transcription pattern indicates morphological and functional chamber characteristics

In both healthy and HCM cats, genes specific for the atrium (*MYL4*, *MYL7* and *NPPA*) and genes specific for the ventricle (*IRX4*, *IRX3*, *MYL2* and *MYL3*) were within the top upregulated genes in the LA and LV samples, respectively. Similarly, higher atrial *NPPA* and ventricular *MYL2* and *MYL3* gene expression was observed in healthy humans [36]. IPA analysis of DEG revealed that most pathways were more activated in the LA compared to the LV, as observed with the gene activation pattern. A higher gene activation in the atria compared to ventricles of healthy cats and cats with HCM has been reported for selected cytokines and extracellular matrix enzymes [34, 35, 38]. Regional differences in gene activation with a clear distinction of atrial and ventricular mRNA expression profiles, and a higher number of DEG in the atria than ventricles, were also found for the healthy human heart [36].

The regional differences in functional pathways are consistent with the morphological characteristics of each chamber and with chamber-specific constitutive physiological and pathological remodeling processes [34–36, 38]. The overall increased number of enriched genes and active pathways in the LA compared to the LV suggests a higher reactivity of the thinner walled LA. The activation of the ERK/MAPK signaling pathway in the healthy LA compared to the LV indicates the ability of the LA to adjust cardiac function to hemodynamic changes [42]. ERK/MAPK is a major pathway in cardiac physiology involved in cell growth and differentiation. Within minutes, MAP kinases (MAPKs) temporarily rectify cardiac output by increasing contractility via cardiomyocyte hypertrophy, for example during exercise [42]. The activation of this pathway in the LA of control cats, together with pathways related to dilated cardiomyopathy signaling, which includes genes specific for cardiac structure and function, β-adrenergic signalling, calcium metabolism and myocardial structure suggest their involvement in LA homeostasis. The genes and corresponding pathways in the healthy LV and LA likely represent a constitutive expression pattern that maintains cardiac structure and function and allows adaptation to hemodynamic changes.

### HCM results in LV and LA specific mRNA transcription profiles, functional pathways and regulators

Comparison of gene expression in HCM vs. the healthy myocardium found more genes to be up- than downregulated in the disease state. Genes related to the sarcomere, remodeling, fibrosis, and inflammatory mediators were upregulated in the HCM LV and LA, with regional differences for specific genes. For the HCM LA, inflammatory cell marker genes were also found to be activated. The top upregulated gene in the HCM LV was thrombospondin 4 (*THBS4*). This gene, which is also one of the top upregulated genes in human HCM hearts [28], is involved in cardiac remodeling and inflammation and associated with interstitial fibrosis [43, 44]. The top upregulated gene in the HCM LA was *FAM177B*, which is a suspected M1 macrophage marker [45] and has been recently identified as a candidate gene for coronary artery disease [46]. Downregulated genes included transcription factors in the HCM LV, and apoptosis and metabolic genes in the LA. *KLHL33* was the top downregulated gene in the HCM LV. Kelch-like proteins are implicated in maintenance of structure and function of organs and in the pathogenesis of various human diseases [47]. *KLHL31*, which is abundant in human and mouse cardiomyocytes, was downregulated in human HCM [28, 47] and *KLHL33* has been reported as relevant locus in Takayasu’s arteritis in humans [48]. The top downregulated gene in the HCM LA was *THRSP*, a gene involved in transcriptional gene regulation and lipogenesis in human liver and adipocytes [49, 50]. The roles of *FAM177B*, *KLHL33* and *THRSP* in the heart are largely unknown and require further investigations. Pathway analysis found structural, fibrosis and inflammatory pathways activated, with some heterogeneity, in the LV and LA of HCM cats, which is similar to what has been reported for human HCM [27–31]. RhoDGI signaling and PPARα/RXRα activation pathways were reduced in the HCM LV. Pathways related to RhoDGI signaling, calcium signaling, opioid signaling and PKA signaling were inhibited in the HCM LA. Predominant upstream regulators were TGFβ1 for the LV and TNFα for the LA. Increased myocardial transcription of these cytokines had been observed in the feline HCM heart previously [10, 34], and both have also been reported as relevant mediators in the human heart [29, 30, 36].

RhoDGI signaling was inhibited in the HCM LV and LA when compared to the healthy counterparts. RhoGDI maintains a stable cytosolic pool of RhoGTPase [51]. RhoGDI reduction might be the cause of RhoFamily GTPase signaling inhibition in the HCM LA and the observed activation of Rac signaling in the HCM LV [51]. RhoGTPAse stimulates downstream signaling of PI3K/Akt, Rac activates PAK and ERK/MAPK pathways [52]. The PI3K/Akt pathway was found to be inhibited in the HCM LA, and PAK and ERK/MAPK pathways were activated in the HCM LV. The cellular responses that are triggered by this signal transduction pathway include an increase in cardiomyocyte size and protein synthesis, actin polymerization and reactivation of fetal genes and stress markers (such as *NPPA*, *NPPB* and *MYH7*) associated with pathological hypertrophy [27, 52, 53]. Both the HCM LV and LA showed activation of pathways related to actin cytoskeleton signaling and cardiac hypertrophy. In the HCM LV, there was upregulation of the actin nucleation pathway [54], the Rac pathway (involved in actin polymerisation), synaptogenesis signaling (indicating actin stabilization) [55] and the glioma signaling pathway. The latter is a GTPase RhoA and Rac mediated pathway that results in cytoskeleton and extracellular matrix remodeling [56, 57]. Cell growth, extracellular matrix, collagen and actin cytoskeleton pathways are also activated in human HCM hearts [25–31]. For the HCM LA, activation of fibrosis pathways was found. *MYH7*, listed within the top upregulated genes in the HCM LA, and *NPPA* in the HCM LV could indicate activation of stress markers and reactivation of fetal genes [25, 27, 52]. RhoGTPase activation is caused by hormones including norepinephrine, endothelin 1, angiotensin II and adrenomedullin, as well as binding to receptors for thrombin, chemokines, cytokines, growth factors and stretch [52, 53, 58]. These hormones and myocardial stretch were likely increased in the HCM study cats, as they were in an advanced stage of disease. Adrenomedullin and thrombin pathways were both activated and the chemokines *CXCL14*, *CXCL6* and *CCL19* as well as the cytokine *IL18* were within the top upregulated genes in the HCM LV when compared to the healthy LV. Similarly, previous studies reported increased myocardial transcription of cytokines, growth factors and extracellular matrix enzymes in cats with HCM [10, 34, 35].

Additional pathways that are involved in cellular proliferation, cardiac hypertrophy, microtubule and cytoskeletal organization, angiogenesis and endothelial function were found to be activated in the HCM samples; their activation is consistent with the histopathology changes in HCM [5, 9–14]. These included the cell surface receptor integrin and ILK signaling [59, 60], reelin signaling mediated by PI3K/Akt, Rho or STAT3 [61, 62], the stathmin 1 pathway (which results in microtubulus stabilization when phosphorylated by PI3K, PKA or MAPK), and the CREB response (which is mediated by RhoGTPases and MAPK) [60]. ILK connects integrins and other cell surface proteins to the actin cytoskeleton, and initiates phosphorylation of protein kinases which are implicated in cardiac cell growth [59, 60]. Our results identified an activation of the ILK pathway in the HCM LV and LA, the integrin and reelin signaling pathways in the LV, and the stathmin 1 and CREB pathways in the LA of HCM cats. Increased transcription of *STAT3* was found in the HCM LV and LA.

RhoGTPase/RhoA-dependent signaling has also been shown to be a key regulator of immune cell differentiation and function. It results in activation of several immune cells including macrophages, dendritic cells, granulocytes, B- and T-cells, and their migration to sites of inflammation [52, 53]. There is also some evidence that RhoA signaling affects macrophage polarization [52, 53]. Classically, M1- and M2-type macrophages are differentiated, with M1-type macrophages being phagocytotic and proinflammatory and releasing reactive oxygen and nitrogen species and cytokines like TNFα, while M2-type macrophages are anti-inflammatory and secrete respective cytokines such as TGFβ and IL10 [53, 63]. Interestingly, the genes for the common macrophage markers *GAL3* and *CD68* were increased in the LV and LA, and for *CD168* also in the LV from HCM cats. In the LA from HCM cats, *FAM177B* (a M1 macrophage marker gene) [45] was the top upregulated gene and TNFα was an upstream regulator, and there was marked activation of the pathway related to production of NO and ROS in macrophages. Furthermore, PKA-induced phosphorylation of RhoA has been reported to inhibit macrophage migration [53]. The reduced PKA signaling in the HCM LA could indicate active macrophage migration. Activation of *FAM177B* and the pathway related to increase of NO and ROS in macrophages imply that M1 macrophages play a role in the LA remodeling process. Furthermore, the T-cell (*CD3D*, *CD3G* and *CD3E*) and B-cell (*CD79*) surface glycoproteins were within the top upregulated genes in the HCM LA, suggesting a potential involvement of T and B lymphocytes. The increase of leukocyte extravasation and acute phase response signaling pathways in the HCM LA indicate an inflammation-favorable environment.

Histopathological and immunohistological investigations reported an increase in interstitial macrophages in the HCM LV [10, 11]. A proliferation of resident macrophages (Iba-1/MHC II positive/calprotectin-negative) was suspected but the type of these macrophages (M1 or M2) was not determined [10, 11]. Only scattered T-cells (CD3-positive) were found. The LA was so far not extensively examined in histological studies. Studies of humans with atrial fibrillation identified an activation of inflammatory factors such as IL6 and TNFα, and an increased number of CD3-positive T cells [64]. Considering that TNFα was identified as an upstream regulator in the HCM LA and IL6 was found to be relevant in HCM (particularly in cats with atrial thrombus) [35], an involvement of T cells in atrial changes in HCM seems likely. For the HCM LV, TGFβ was identified as an upstream regulator and the M2 macrophage gene *IL10* was upregulated when compared with the healthy LV. However, no differences were found for the M2 markers *EGR2* and *MYC*, and the transcription of the M1 marker *GALS3BP* was increased in the HCM LV. This suggests that both types of macrophages (M1 and M2) are present in the HCM LV.

Further involved are the LXR/RXR and PPARα/RXRα pathways. The LXR/RXR pathway was activated in the HCM LA, whilst PPARα/RXRα activation was reduced in the HCM LV when compared with the healthy counterpart. The LXR/RXR pathway attenuates cardiomyocyte hypertrophy, promotes cell survival, modulates myocardial metabolism, has anti-inflammatory effects, promotes angiogenesis, and inhibits fibrosis [65, 66]. A large animal model of pacing-induced cardiomyopathy showed inhibition of the LXR/RXR pathway, activation of the ILK pathway, and increased inflammatory cytokines, apoptosis and fibrosis [67]. LXR/RXR pathway activation in the HCM LA might therefore indicate cardioprotective effects.

PPARα/RXRα regulates energy metabolism. It acts as a fatty acid sensor and promotes fatty acid catabolism, inhibits gluconeogenesis, and reduces ATP synthesis. It has anti-inflammatory effects and suppresses angiogenesis [68–70]. The reduction of this pathway in the LV supports the presence of an inflammatory environment, metabolic changes and new vessel formation [10, 11, 25, 26, 30, 34, 35]. This finding is consistent with the activated HIF1α and CXCR4 pathways in the HCM LV [71, 72].

HIF1α, which is induced in response to hypoxia and inflammatory cytokines, regulates the expression of multiple common genes such as CXCR4, adrenomedullin pathways, and Glut-1 [73], which were all found to be higher in the HCM LV than the healthy LV. These result in angiogenesis, glycolysis, cell proliferation and survival, and promote anerobic metabolism [71, 72, 74]. CXCR4 is a chemokine receptor that is expressed in cardiomyocytes, fibroblasts and endothelial cells, mediates homing of progenitor cells, is involved in cell proliferation, migration and survival; and has been identified as a hub gene in human HCM [26, 71, 72, 75, 76]. In a mouse model of heart failure with preserved ejection fraction, increased CXCR4-positive macrophages were found in the circulation and the myocardium, which was associated with myofibroblast differentiation, fibrosis and an increased in inflammatory cytokines [77]. The gene expression for the chemokines *MIF* and *CXCL12*, which are the main CXCR4 ligands, were reduced in the HCM LV. This might result in fewer inflammatory monocytes being recruited and supports a potential predominance of anti-inflammatory macrophages in the feline HCM LV [71, 72]. The observed gene expression, the reduction of PPARα/RXRα, and the activation of HIF1α, CXCR4 and adrenomedullin pathways in the feline HCM LV could result in angiogenesis and vasodilation improving tissue hypoxia, stem cell mobilization and cell survival, and could be the reason that morbidity and death pathways were reduced in the IPA network analysis. These findings are supported by previous studies that identified attempts of angiogenesis, increased endothelial nitric oxide synthase mRNA and the presence of CD34-positive cells (which indicate a degree of stemness) in the feline HCM LV [10, 11].

The calcium signaling pathway was found to be inhibited in the LA of HCM hearts. This is in accordance with the reduced endocannabinoid neuronal synapse and opioid signaling pathways [78, 79]. Both signaling pathways have several downstream effects including inhibiting calcium influx, PKA and PI3k/Akt pathways and stimulating CREB signaling pathways [78, 79], which were also observed for the HCM LA. The relevance of calcium and these pathways for cardiac structure and function is well known and clustering of gene expression from human patients found calcium signaling and conduction as one module consistent with HCM-related processes [27].

Limitations to this study include the lack of accounting for age-associated cardiac changes in gene expression. For LXL/RXR in humans and mouse models as well as for myocardial cytokine and remodeling marker transcription in healthy cats and cats with HCM, an influence of age was reported [10, 38, 66]. However, the young control cats represent a homogenous control group that allowed the identification of the constitutive gene transcription in the healthy cat heart. We work with pet cats and not with animal models; for ethical reasons, healthy adult cats are not euthanized, so such hearts are rarely available. Euthanasia of adult cats is carried out for medical reasons, and these cats suffer from progressed (systemic) diseases that influence myocardial gene transcription and would preclude the differentiation from gene activation observed with HCM [34]. The cats with HCM had progressed disease, and therefore the gene expression patterns might not reflect those involved in early stages of the disease and might be influenced by heart failure [80]. Echocardiography of cats with HCM was obtained at different time points prior to euthanasia. These data do not reflect the stage of heart disease at the time point of death and were therefore not included into the study. Hearts from the young control cats were donated and echocardiography was not part of their assessment, although no cardiac abnormalities were observed on gross and histopathological examination. The sample size was small, which was mainly caused by the requirement of sampling the hearts within 30 minutes after death to ensure adequate RNA quality. However, the sample size was sufficient for the sequencing analysis and the statistical power was increased by the inclusion of paired (matched) tissue samples from the 5 healthy control cats and 3 individual HCM hearts.

## Conclusions

The present study is the first to report global differences in cardiac gene expression that differentiates by region (LV vs. LA) and by health status (HCM vs. healthy) applying RNA-seq. Identification of DEG, their function and associated pathways, regulators and networks revealed unique gene expression patterns in the LV and LA regions of the cat heart that contribute to the distinct structure and function of each chamber in health and HCM. Similarities were observed to the DEG and associated functional pathways reported for human HCM. The top enriched gene in the HCM LV, *THBS4*, is also top enriched in the human HCM myocardium. Interestingly, the top DEG *KLHL33* (LV), *FAM177B* and *THRSP* (LA) have not been reported for the heart so far, and their role in the heart and in HCM requires further investigation. These differences in DEG to human HCM are likely caused by different disease stages and heterogeneity across hearts [26, 27, 30, 81] and might be associated with the observed variability in clinical presentation. RhoGDI-RhoGTPase signaling seems to play a central role in the remodeling processes in feline HCM. Genes and pathways associated with structural remodeling and inflammation were activated in the HCM LV and LA, exhibiting some regional differences. Fibrosis and calcium signaling with infiltration of inflammatory cells seem to be of relevance in the HCM LA disease process, while in the LV, an inflammatory environment, microvascular changes, and adaptation to hypoxia seem to predominate. Other findings that might be of particular interest for disease processes occurring in HCM include the role of integrin, ILK signaling, and the LXR/RXR pathway in the LA, and the PPARα/RXRα, HIF1α and CXCR4 pathways in the LV. The present study is of high relevance since knowledge of the pathogenesis of feline and human HCM is still fragmentary, and since mouse models generally fall short for research into HCM [82]. Studying the naturally occurring disease in cats, a species that has been suggested as a model for human HCM [4–6], and not in experimental models is the strength of our research, and DEG and pathways previously not considered in cardiac pathology, particularly in HCM, were identified.

## Supporting information

S1 Table Primers used for reverse transcription quantitative polymerase chain reaction. *ANKRD2*: ankyrin repeat domain 2, *CXCL14*: C-X-C Motif Chemokine Ligand 14, *CXCL6*: C-X-C Motif Chemokine Ligand 6, *FAM177B*: Family with Sequence Similarity 177 Member B, *GAPDH*: glyceraldehyde-3-phosphate dehydrogenase, *ID3*: Inhibitor of DNA binding 3, *IL18*: Interleukin 18, *RPS7*: Ribosomal Protein S7, *THBS4*: Thrombospondin 4.
(DOCX)

S2 Table Top differently expressed genes in the left ventricle of healthy cats compared to the left atrium of healthy cats. **A**. Top upregulated genes in the left ventricle of healthy cats compared to the left atrium of healthy cats. **B**. Top downregulated genes in the left ventricle of healthy cats compared to the left atrium of healthy cats.
(DOCX)

S3 Table Top differently expressed genes in the left ventricle of HCM cats compared to the left atrium of HCM cats. **A**. Top upregulated genes in the left ventricle of HCM cats compared to the left atrium of HCM cats. **B**. Top downregulated genes in the left ventricle of HCM cats compared to the left atrium of HCM cats.
(DOCX)

S4 Table Top differently expressed genes in the left ventricle of HCM cats compared to the left ventricle of healthy cats. **A**. Top upregulated genes in the left ventricle of HCM cats compared to the left ventricle of healthy cats. **B**. Top downregulated genes in the left ventricle of HCM cats compared to the left ventricle of healthy cats.
(DOCX)

S5 Table Top differently expressed genes in the left atrium of HCM cats compared to the left atrium of healthy cats. **A**. Top upregulated genes in the left atrium of HCM cats compared to the left atrium of healthy cats. **B**. Top downregulated genes in the left atrium of HCM cats compared to the left atrium of healthy cats.
(DOCX)
