# Supplementary material for: Feline myocardial transcriptome in health and in hypertrophic cardiomyopathy—A translational animal model for human disease
Source: PLoS One. 2023 Mar 16;18(3):e0283244. doi: 10.1371/journal.pone.0283244 (PMC10019628; doi:10.1371/journal.pone.0283244)
Supplement: S1 Table — ANKRD2: ankyrin repeat domain 2, CXCL14: C-X-C Motif Chemokine Ligand 14, CXCL6: C-X-C Motif Chemokine Ligand 6, FAM177B: Family with Sequence Similarity 177 Member B, GAPDH: glyceraldehyde-3-phosphate dehydrogenase, ID3: Inhibitor of DNA binding 3, IL18: Interleukin 18, RPS7: Ribosomal Protein S7, THBS4: Thrombospondin 4. (DOCX) [file pone.0283244.s001.docx]

**S1 Table. Primers used for reverse transcription quantitative polymerase chain reaction.**

| Genes | Primer direction | Sequence (5’-3’) | Product length (nucleotides) |
| --- | --- | --- | --- |
| ANKRD2 | Forward | GCTCCGAGTCTGGCCTTACA | 20 |
|  | Reverse | CGCCGAAGGTCTAGTGATGTCT | 22 |
| CXCL14 | Forward | GACGTGAAGAAGCTGGAAATGAA | 23 |
|  | Reverse | ACGCTCTTGGTGGTGATGATAAC | 23 |
| CXCL6 | Forward | TGCTGTGCTATTGAAACCTTTGG | 23 |
|  | Reverse | CCTTGAACGATTAACAACCAGTGA | 24 |
| THBS4 | Forward | TGCACCCAAGTGGATTCTGTT | 21 |
|  | Reverse | TGGACTCAGGACTCTGGGAAGA | 22 |
| ID3 | Forward | GGCTCTGTTGCCCTGATTATGA | 22 |
|  | Reverse | TTGCATGGTTACAGAAAGTCACCT | 24 |
| IL18 | Forward | TTTGTAGCTGACAGTGATGAAAACC | 25 |
|  | Reverse | CAGGTTGATCTCCCTGGTTAATG | 23 |
| GAPDH | Forward | GCCATCAATGACCCCTTCAT | 20 |
|  | Reverse | GCCGTGGAATTTGCCGT | 17 |
| RPS7 | Forward | GTCCCAGAAGCCGCACTTT | 19 |
|  | Reverse | CACAATCTCGTCTGGGAAAA | 20 |

*ANKRD2*: ankyrin repeat domain 2, *CXCL14*: C-X-C Motif Chemokine Ligand 14, *CXCL6*: C-X-C Motif Chemokine Ligand 6, *FAM177B*: Family with Sequence Similarity 177 Member B, *GAPDH*: glyceraldehyde-3-phosphate dehydrogenase, *ID3*: Inhibitor of DNA binding 3, *IL18*: Interleukin 18, *RPS7*: Ribosomal Protein S7, *THBS4*: Thrombospondin 4.
