# Supplementary material for: Feline myocardial transcriptome in health and in hypertrophic cardiomyopathy—A translational animal model for human disease
Source: PLoS One. 2023 Mar 16;18(3):e0283244. doi: 10.1371/journal.pone.0283244 (PMC10019628; doi:10.1371/journal.pone.0283244)
Supplement: S3 Table — A. Top upregulated genes in the left ventricle of HCM cats compared to the left atrium of HCM cats. B. Top downregulated genes in the left ventricle of HCM cats compared to the left atrium of HCM cats. (DOCX) [file pone.0283244.s003.docx]

**S3A Table. Top upregulated genes in the left ventricle of HCM cats compared to the left atrium of HCM cats.**

| Ensemble ID | Gene Coordinate | Gene version | Gene Name | Gene Type | LogFC | Adjusted p-value |
| --- | --- | --- | --- | --- | --- | --- |
| ENSFCAG00000022359 | A1:240951021-240960371 | 3 | IRX4 | protein_coding | 7.28 | 4.57E-12 |
| ENSFCAG00000013825 | D1:83411606-83460444 | 5 | SLC5A12 | protein_coding | 6.25 | 2.51E-06 |
| ENSFCAG00000038136 | A2:30850745-31165839 | 2 | SYNPR | protein_coding | 5.45 | 8.55E-05 |
| ENSFCAG00000026641 | E1:62220936-62222172 | 3 | PPP1R27 | protein_coding | 4.69 | 8.55E-05 |
| ENSFCAG00000043976 | F2:4092186-4095049 | 1 | PPDPFL | protein_coding | 4.48 | 4.05E-09 |
| ENSFCAG00000041752 | E2:34545304-34550695 | 1 | IRX5 | protein_coding | 4.44 | 1.65E-05 |
| ENSFCAG00000039768 | E2:33961955-33964313 | 2 | IRX3 | protein_coding | 4.21 | 3.94E-06 |
| ENSFCAG00000005515 | D4:75435449-75436021 | 3 | RNF183 | protein_coding | 3.88 | 4.83E-05 |
| ENSFCAG00000046607 | A3:15504364-15506088 | 1 | WFDC11 | protein_coding | 3.87 | 3.03E-11 |
| ENSFCAG00000011798 | E2:26891324-26919364 | 5 | SHCBP1 | protein_coding | 3.82 | 2.20E-06 |
| ENSFCAG00000018105 | C1:106829994-106839447 | 4 | RORC | protein_coding | 3.70 | 8.20E-07 |
| ENSFCAG00000012799 | C1:19577766-19593398 | 5 | EXTL1 | protein_coding | 3.67 | 5.15E-04 |
| ENSFCAG00000028244 | B3:48590294-48637829 | 3 | AQP9 | protein_coding | 3.64 | 1.31E-09 |
| ENSFCAG00000042774 | D2:34531280-34531648 | 1 | C10orf105 | protein_coding | 3.52 | 4.17E-09 |
| ENSFCAG00000014815 | C2:661625-681496 | 5 | FTCD | protein_coding | 3.41 | 3.19E-05 |
| ENSFCAG00000040762 | B3:140963736-140963882 | 1 | TUNAR | protein_coding | 3.40 | 1.53E-04 |
| ENSFCAG00000024574 | C2:79153302-79291085 | 3 | OSTN | protein_coding | 3.38 | 1.10E-05 |
| ENSFCAG00000001840 | E1:4224221-4240786 | 5 | DHRS7C | protein_coding | 3.23 | 1.11E-03 |
| ENSFCAG00000002673 | C1:95807545-96011630 | 5 | KCND3 | protein_coding | 3.13 | 2.23E-04 |
| ENSFCAG00000011093 | B2:5541844-5608641 | 5 | GPLD1 | protein_coding | 3.12 | 4.77E-05 |
| ENSFCAG00000005244 | F2:32090263-32101158 | 4 | PMP2 | protein_coding | 3.08 | 2.64E-03 |
| ENSFCAG00000031739 | D1:61854318-61858213 | 3 | ART5 | protein_coding | 3.01 | 2.68E-07 |
| ENSFCAG00000024280 | A2:79037401-79038702 | 3 | GPR22 | protein_coding | 2.99 | 4.84E-05 |
| ENSFCAG00000029751 | F2:85419035-85423693 | 3 | GPT | protein_coding | 2.93 | 1.77E-09 |
| ENSFCAG00000031838 | D1:61865830-61869785 | 3 | ART1 | protein_coding | 2.90 | 1.46E-08 |

**S3B Table. Top downregulated genes in the left ventricle of HCM cats compared to the left atrium of HCM cats.**

| Ensemble ID | Gene Coordinate | Gene version | Gene Name | Gene Type | LogFC | Adjusted p-value |
| --- | --- | --- | --- | --- | --- | --- |
| ENSFCAG00000014360 | A2:62431083-62433496 | 5 | MYL7 | protein_coding | -8.70 | 2.86E-11 |
| ENSFCAG00000003878 | E1:46386618-46416387 | 4 | MYL4 | protein_coding | -8.49 | 7.38E-17 |
| ENSFCAG00000018217 | B4:133085738-133102708 | 4 | PVALB | protein_coding | -8.11 | 1.27E-06 |
| ENSFCAG00000026484 | C2:65418872-65449039 | 3 | UPK1B | protein_coding | -8.11 | 1.31E-05 |
| ENSFCAG00000004847 | B3:3751687-3782920 | 4 | BNC1 | protein_coding | -8.05 | 2.24E-06 |
| ENSFCAG00000004394 | E3:6010411-6022430 | 4 | NPTX2 | protein_coding | -8.00 | 8.91E-12 |
| ENSFCAG00000009978 | B4:92455520-92525076 | 5 | WIF1 | protein_coding | -6.88 | 2.34E-07 |
| ENSFCAG00000001198 | A3:32516969-32527382 | 4 | LRRN4 | protein_coding | -6.58 | 4.47E-06 |
| ENSFCAG00000044394 | E3:44471375-44472168 | 1 | HBM | protein_coding | -6.42 | 2.23E-03 |
| ENSFCAG00000028303 | E2:54186891-54195272 | 3 | CLEC3A | protein_coding | -6.31 | 3.56E-05 |
| ENSFCAG00000000299 | B4:81398354-81404817 | 5 | KRT18 | protein_coding | -6.16 | 1.38E-05 |
| ENSFCAG00000033577 | B3:147342083-147345095 | 2 | LBHD2 | protein_coding | -5.95 | 1.24E-03 |
| ENSFCAG00000023364 | E2:45916956-45930708 | 3 | SMPD3 | protein_coding | -5.38 | 6.88E-05 |
| ENSFCAG00000000270 | B4:80751042-80765014 | 4 | KRT7 | protein_coding | -5.30 | 3.11E-05 |
| ENSFCAG00000022358 | B1:42285303-42301317 | 3 | CHRNA6 | protein_coding | -5.16 | 1.73E-05 |
| ENSFCAG00000000072 | C1:8668478-8678472 | 4 | NPPA | protein_coding | -5.14 | 2.91E-11 |
| ENSFCAG00000009754 | A2:13900923-13909129 | 5 | COMP | protein_coding | -5.07 | 4.84E-05 |
| ENSFCAG00000008974 | B3:6074208-6093201 | 5 | ANPEP | protein_coding | -5.04 | 1.81E-04 |
| ENSFCAG00000000298 | B4:81356467-81364065 | 4 | KRT8 | protein_coding | -4.95 | 1.14E-07 |
| ENSFCAG00000006143 | C2:151423941-151429898 | 4 | CCK | protein_coding | -4.89 | 2.84E-03 |
| ENSFCAG00000011926 | D3:22744933-22825862 | 5 | HORMAD2 | protein_coding | -4.88 | 7.57E-07 |
| ENSFCAG00000002641 | E1:37729413-37735332 | 4 | TAC4 | protein_coding | -4.86 | 7.93E-06 |
| ENSFCAG00000010009 | B3:147353622-147362241 | 5 | EXOC3L4 | protein_coding | -4.74 | 2.86E-07 |
| ENSFCAG00000008761 | C1:29149900-29167424 | 5 | RSPO1 | protein_coding | -4.64 | 3.48E-06 |
| ENSFCAG00000013683 | D1:107765820-107799578 | 5 | MYRF | protein_coding | -4.47 | 8.52E-08 |
