# Supplementary material for: Feline myocardial transcriptome in health and in hypertrophic cardiomyopathy—A translational animal model for human disease
Source: PLoS One. 2023 Mar 16;18(3):e0283244. doi: 10.1371/journal.pone.0283244 (PMC10019628; doi:10.1371/journal.pone.0283244)
Supplement: S4 Table — A. Top upregulated genes in the left ventricle of HCM cats compared to the left ventricle of healthy cats. B. Top downregulated genes in the left ventricle of HCM cats compared to the left ventricle of healthy cats. (DOCX) [file pone.0283244.s004.docx]

**S4A Table.** **Top upregulated genes in the left ventricle of HCM cats compared to the left ventricle of healthy cats.**

| Ensemble ID | Gene Coordinate | Gene version | Gene Name | Gene Type | LogFC | Adjusted p-value |
| --- | --- | --- | --- | --- | --- | --- |
| ENSFCAG00000003879 | A1:146252507-146296122 | 5 | THBS4 | protein_coding | 5.67 | 1.67E-05 |
| ENSFCAG00000040779 | B2:35269022-35291521 | 1 | IP6K3 | protein_coding | 5.29 | 3.11E-05 |
| ENSFCAG00000038136 | A2:30850745-31165839 | 2 | SYNPR | protein_coding | 4.83 | 2.27E-04 |
| ENSFCAG00000014088 | B3:115769845-115801118 | 4 | ARG2 | protein_coding | 4.53 | 5.49E-05 |
| ENSFCAG00000010232 | B3:139594825-139603785 | 4 | SERPINA1 | protein_coding | 4.26 | 1.33E-04 |
| ENSFCAG00000036039 | C1:196289445-196328114 | 2 | MYL1 | protein_coding | 4.12 | 6.53E-05 |
| ENSFCAG00000026148 | A2:117103787-117129621 | 3 | GPNMB | protein_coding | 4.08 | 7.38E-05 |
| ENSFCAG00000004938 | D4:76919625-77015039 | 5 | TNC | protein_coding | 3.99 | 9.28E-05 |
| ENSFCAG00000025457 | B1:173311731-173327417 | 3 | UCHL1 | protein_coding | 3.88 | 1.79E-07 |
| ENSFCAG00000013929 | A1:114455813-114463990 | 5 | CXCL14 | protein_coding | 3.87 | 1.10E-06 |
| ENSFCAG00000006366 | B3:121878578-121984509 | 5 | LTBP2 | protein_coding | 3.62 | 2.75E-07 |
| ENSFCAG00000026846 | D4:1673520-1677115 | 3 | BARX1 | protein_coding | 3.61 | 2.98E-04 |
| ENSFCAG00000000072 | C1:8668478-8678472 | 4 | NPPA | protein_coding | 3.60 | 3.71E-09 |
| ENSFCAG00000026644 | A2:5985490-5985738 | 3 | CTXN1 | protein_coding | 3.54 | 4.30E-06 |
| ENSFCAG00000014969 | B1:148041165-148042272 | 4 | CXCL6 | protein_coding | 3.49 | 1.79E-05 |
| ENSFCAG00000003162 | D4:58501171-58502989 | 4 | CCL19 | protein_coding | 3.42 | 1.23E-03 |
| ENSFCAG00000029199 | D1:10128311-10147653 | 3 | IL18 | protein_coding | 3.38 | 3.34E-07 |
| ENSFCAG00000013428 | D2:58762184-58771951 | 5 | ANKRD2 | protein_coding | 3.34 | 6.48E-04 |
| ENSFCAG00000002648 | X:110604600-110621340 | 4 | IGSF1 | protein_coding | 3.31 | 9.16E-06 |
| ENSFCAG00000000329 | E2:7742117-7768273 | 5 | MYBPC2 | protein_coding | 3.26 | 4.24E-06 |
| ENSFCAG00000031151 | F1:43957682-43968886 | 3 | FMOD | protein_coding | 3.26 | 6.80E-05 |
| ENSFCAG00000010181 | C1:12208353-12252105 | 4 | PADI2 | protein_coding | 3.23 | 8.79E-07 |
| ENSFCAG00000004465 | C1:201985533-202011512 | 4 | IGFBP2 | protein_coding | 3.12 | 7.08E-06 |
| ENSFCAG00000024574 | C2:79153302-79291085 | 3 | OSTN | protein_coding | 3.12 | 4.76E-04 |
| ENSFCAG00000043407 | B1:150687483-150695920 | 1 | JCHAIN | protein_coding | 2.99 | 6.19E-04 |

**S4B Table. Top downregulated genes in the left ventricle of HCM cats compared to the left ventricle of healthy cats.**

| Ensemble ID | Gene Coordinate | Gene version | Gene Name | Gene Type | LogFC | Adjusted p-value |
| --- | --- | --- | --- | --- | --- | --- |
| ENSFCAG00000006930 | B3:73585522-73594199 | 5 | KLHL33 | protein_coding | -3.68 | 1.99E-05 |
| ENSFCAG00000029636 | F1:32916374-32930180 | 3 | ATF3 | protein_coding | -3.14 | 1.29E-03 |
| ENSFCAG00000042161 | A2:23681625-23704218 | 1 | WNT5A | protein_coding | -3.03 | 1.27E-07 |
| ENSFCAG00000000153 | B3:27381201-27877048 | 5 | OCA2 | protein_coding | -2.78 | 2.59E-04 |
| ENSFCAG00000011976 | D4:49513305-49680415 | 4 | ELAVL2 | protein_coding | -2.63 | 4.15E-04 |
| ENSFCAG00000001812 | B2:124810120-124844849 | 5 | MYB | protein_coding | -2.48 | 1.94E-04 |
| ENSFCAG00000005892 | D1:91193976-91269313 | 5 | PAMR1 | protein_coding | -2.47 | 1.60E-04 |
| ENSFCAG00000040059 | B2:22263498-22324782 | 2 | LY86 | protein_coding | -2.42 | 2.26E-05 |
| ENSFCAG00000013175 | A2:12389959-12451628 | 5 | NWD1 | protein_coding | -2.30 | 6.41E-04 |
| ENSFCAG00000012668 | E3:9993287-10002811 | 4 | TRIM50 | protein_coding | -2.14 | 3.79E-06 |
| ENSFCAG00000031739 | D1:61854318-61858213 | 3 | ART5 | protein_coding | -2.14 | 6.15E-06 |
| ENSFCAG00000035352 | D1:25852858-25882749 | 2 | KCNJ5 | protein_coding | -2.12 | 8.08E-04 |
| ENSFCAG00000033652 | A3:49474808-49578302 | 2 | SYNDIG1 | protein_coding | -2.02 | 1.14E-04 |
| ENSFCAG00000031965 | C1:72499633-72502604 | 3 | CCN1 | protein_coding | -1.99 | 1.99E-05 |
| ENSFCAG00000023685 | A3:56832584-56844838 | 3 | C2orf40 | protein_coding | -1.96 | 8.15E-06 |
| ENSFCAG00000000214 | A2:124634700-125173889 | 5 | PDE1C | protein_coding | -1.95 | 2.48E-05 |
| ENSFCAG00000005336 | C2:130562616-130609183 | 5 | KY | protein_coding | -1.94 | 6.78E-05 |
| ENSFCAG00000003989 | C1:44720001-44750517 | 4 | DHCR24 | protein_coding | -1.89 | 1.55E-04 |
| ENSFCAG00000002555 | E1:60147844-60330761 | 5 | RBFOX3 | protein_coding | -1.84 | 8.63E-05 |
| ENSFCAG00000011244 | A2:51427968-51432910 | 4 | GHRL | protein_coding | -1.83 | 2.01E-04 |
| ENSFCAG00000034483 | B4:126717689-126985196 | 2 | CHST11 | protein_coding | -1.78 | 9.71E-05 |
| ENSFCAG00000007029 | D1:74896340-75117750 | 5 | PLEKHA7 | protein_coding | -1.73 | 4.45E-08 |
| ENSFCAG00000011288 | F1:25199497-25219865 | 5 | IVNS1ABP | protein_coding | -1.68 | 1.58E-05 |
| ENSFCAG00000006729 | F1:46081663-46113447 | 5 | PM20D1 | protein_coding | -1.64 | 4.48E-04 |
| ENSFCAG00000028457 | C2:95783571-95843209 | 3 | NCEH1 | protein_coding | -1.64 | 1.22E-06 |
